# Antenna Allocation in MIMO Radar with Widely Separated Antennas for Multi-Target Detection

**DOI:** 10.3390/s141120165

**Published:** 2014-10-27

**Authors:** Hao Gao, Jian Wang, Chunxiao Jiang, Xudong Zhang

**Affiliations:** Department of Electronic Engineering, Tsinghua University, Beijing 100084, China; E-Mails: h-gao10@mails.tsinghua.edu.cn (H.G.); jian-wang@tsinghua.edu.cn (J.W.); jchx@tsinghua.edu.cn (C.J.)

**Keywords:** relative entropy, MIMO radar, antenna allocation, multi-target detection

## Abstract

In this paper, we explore a new resource called multi-target diversity to optimize the performance of multiple input multiple output (MIMO) radar with widely separated antennas for detecting multiple targets. In particular, we allocate antennas of the MIMO radar to probe different targets simultaneously in a flexible manner based on the performance metric of relative entropy. Two antenna allocation schemes are proposed. In the first scheme, each antenna is allocated to illuminate a proper target over the entire illumination time, so that the detection performance of each target is guaranteed. The problem is formulated as a minimum makespan scheduling problem in the combinatorial optimization framework. Antenna allocation is implemented through a branch-and-bound algorithm and an enhanced factor 2 algorithm. In the second scheme, called antenna-time allocation, each antenna is allocated to illuminate different targets with different illumination time. Both antenna allocation and time allocation are optimized based on illumination probabilities. Over a large range of transmitted power, target fluctuations and target numbers, both of the proposed antenna allocation schemes outperform the scheme without antenna allocation. Moreover, the antenna-time allocation scheme achieves a more robust detection performance than branch-and-bound algorithm and the enhanced factor 2 algorithm when the target number changes.

## 1. Introduction

Multiple input multiple output (MIMO) radar has received considerable attention in recent years. Unlike a standard phased-array radar, where different antennas transmit the scaled and phase-shifted version of the same waveform, MIMO radar can transmit different waveforms at different antennas [1]. In general, MIMO radars can be classified into two categories—MIMO radar with colocated antennas [1] and MIMO radar with widely separated antennas [2]. MIMO radar with colocated antennas improves parameter estimation and beamforming performance by having more effective spatial degrees of freedom [3,4], since its transmit antennas and receive antennas are close enough to observe coherent signals reflected from the target. MIMO radar with widely separated antennas, also referred to as statistical MIMO radar, improves detection and estimation resolution by exploiting the diversity of the propagation path [5,6]. In this paper, we are concerned with a MIMO radar with widely separated antennas, which can be viewed as a type of multistatic radar. Each antenna of the MIMO radar can steer its beam independently towards any direction as an independent transmitter. However, this system differs from the multistatic radar by emphasizing the joint processing of signals for transmission and reception [2].

There are many resource optimization problems we could consider to improve the performance of MIMO radar. Waveform design is one of the most interesting resource optimization problems [7–17]. Optimal illumination cooperation from MIMO radar waveform design can further enhance the capabilities of the radar system. With limited total transmit power, power allocation is regarded as another important radar resource optimization problem [6,18]. In addition, antenna allocation is also analyzed as a resource optimization problem for parameter estimation [19,20]. In order to select an appropriate antenna subset for single-target localization, antenna allocation was formulated as a knapsack problem in [19]. Other formulations of selecting sensors could also be found in [20–22]. However, these researches focus on the optimization of the resource for a single target. In fact, there is another type of resource that we could use to further optimize the performance of MIMO radar: multi-target diversity. We consider a multi-static radar, where its transmit and receive antennas are spaced far away. Furthermore, we assume each transmit antenna is able to steer its beam independently towards different targets and that the receive antennas are able to receive all of the signals reflected from different targets. Therefore, the entire set of antennas form a statistical MIMO radar. We expect that, by steering each antenna to illuminate the “most proper” target, we could improve the overall detection performance. Current literature on multiple targets is not related to the idea of multi-target diversity in the statistical MIMO radar. In [23] and [24], energy allocation for detecting multiple targets in mono-static radar was considered. Additionally, multi-target localization in MIMO radar was investigated in [25,26]. To the best of our knowledge, antenna allocation of a statistical MIMO radar for multi-target detection has not been addressed so far.

Metrics have been introduced to measure the performance of MIMO radar, and these can be used as criteria to optimize the resources. Information theoretic criterion, which was first introduced to radar receiver design in [27], has received considerable attention. Mutual information criterion was proposed for radar waveform design in [8–14]. By maximizing the mutual information between target impulse response and target echoes, radar systems could improve estimation and detection performance. The minimum mean square error (MMSE) criterion was also introduced in [9], which led to the same estimation result as the mutual information criterion. Focusing the transmit power in the target direction was another way to improve estimation accuracy [3,15,16]. Minimizing the probability of miss detection for a given probability of false alarm and maximizing the signal-to-interference were proposed in [11] and [17], respectively. In [14] and [28], another information theoretic criterion, *i.e.*, relative entropy, was used to study the detection performance. It originated from Stein's lemma [29]. Relative entropy is capable of approximately characterizing the probability of a miss in target detection, while mutual information is incapable. Therefore, we will employ relative entropy to analyze radar detection performance in this paper.

Based on relative entropy, the multi-target detection problem can be formulated as a minimum makespan scheduling problem in the combinatorial optimization framework [30]. Minimum makespan scheduling problems are classified as non-deterministic polynomial-time complete ( 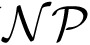-complete) in the strong sense [31]. Exhaustive search can be used to solve these problems. This algorithm is simple and always leads to a solution. However, the computational complexity will exponentially grow as the problem size increases. A solution with a given error to the minimum makespan scheduling problem was shown in [30]. Other polynomial-time approximation schemes were also proposed with little penalty compared to an exhaustive search. In [31], the author proposed a polynomial-time approximation algorithm in identical machines, and in [32], the author considered related machines rather than identical machines. However, the “machines” in our problem may be totally different. Therefore, new algorithms will be used in our problem.

The main contributions of this paper are as follows.

1. Antenna allocation is introduced to exploit the “multi-target diversity” to enhance the detection performance. To implement antenna allocation, a statistical MIMO radar is employed to illuminate multiple targets simultaneously. Two comparative schemes—time allocation and uniform allocation—are also used in the experiments. These two schemes both sequentially illuminate targets one by one.
2. The antenna allocation problem is formulated as a minimum makespan scheduling problem in a combinatorial optimization framework. In the antenna allocation problem, the detection performance is characterized by relative entropy. The contributions of antennas can be effectively modeled as “processing times” in makespan, and target cells can be effectively modeled as “machines”, which are not identical. A branch-and-bound algorithm is used to achieve the optimal antenna allocation result through a simple transformation of the original problem. Moreover, a new antenna allocation algorithm, called the enhanced factor 2 algorithm, is proposed. This heuristic algorithm employs a greedy strategy to allocate antennas for multiple targets. Both of the branch-and-bound algorithm and the enhanced factor 2 algorithm just allocate each antenna to illuminate a certain fixed target over the entire illumination time. Therefore, the branch-and-bound algorithm and enhanced factor 2 algorithm can be regarded as an antenna-only allocation scheme.
3. More importantly, we propose an antenna-time allocation scheme as an alternative to the 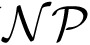-hard antenna allocation problem. Using the antenna-time allocation scheme, each transmit antenna is selected to illuminate different targets with different illumination time. Therefore, each target has an opportunity to be illuminated by all transmit antennas, so that a new proper antenna allocation result can be explored. In fact, the antenna-time allocation scheme can be regarded as an extension of the antenna-only allocation scheme, since it optimizes both antenna allocation and illumination time allocation.

The rest of this paper is organized as follows. The signal model of MIMO radar and the multi-target detection problem are introduced in Section 2. Antenna allocation schemes are proposed in Section 3, including the antenna-only allocation scheme and the antenna-time allocation scheme. Numerical experiments and analysis are shown in Section 4. Finally, Section 5 concludes the paper.

## 2. Problem Formulation

### 2.1. Signal Model

Assume that the entire search area of the space is divided into *K* target cells, where each cell contains either one or zero targets. Whether there is a target in a certain cell is independent of that in other cells. We consider a MIMO radar consisting of *M* transmit antennas and *N* receive antennas. Each transmit antenna is able to steer its beam independently to illuminate any of the target cells. Denote the entire set of transmit antennas by a set 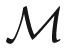 = {1,…, *M*}. Split 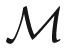 into *K* non-overlapping subsets of antennas, and let 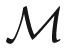
*_k_* ⊆ 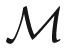 denote the *k*-th subset, which illuminates target *k*. Let *M_k_* denote the cardinality of the set 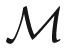
*_k_*. We assume that each antenna illuminates one target cell, so that $\sum_{k=1}^{K}M_{k}=M$. In realistic scenarios, target number *K* can be very large in the entire surveillance region. Therefore, we have to detect several times to cover the entire region. For each time of detection, we assume that the number of targets is less than the number of transmit antennas, *i.e.*, *M* > *K*. Denote the waveform of the *m*-th transmit antenna by a vector *s_m_*. Thus, the transmitted waveform for target cell *k* is denoted by a matrix ***S****_k_* = [***s***_*k*__1___, ***s***_*k*__2___, ⋯, ***s****_k_M_k___*], where the subscript *k*_1_ ∈ 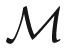
*_k_* denotes the first transmit antenna in 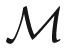
*_k_* steered towards target *k*. The transmitted waveform is assumed to be narrowband. Therefore, the waveform that arrives at the *n*-th receiver can be modeled as [2,7]:

(1) $${\text{r}}_{k,n}={\text{S}}_{k}h_{k,n}$$

where ***h****_k_*_,_*_n_* = [*h*_1,_*_k_*_,_*_n_*,*h*_2,_*_k_*_,_*_n_*, ⋯, *h_M_k__*_,_*_k_*_,_*_n_*]*^T^* is a *M_k_* × 1 vector, which denotes the *k*-th target scattering coefficients for receiver *n*, and each element *h_m_*_,_*_k_*_,_*_n_* in ***h****_k_*_,_*_n_* denotes the scattering coefficient from the *m*-th transmitter to the *n*-th receiver for the *k*-th target. Let ***R****_k_* denote the matrix that collects ***r****_k_*_,_*_n_* at different receivers (*n* = 1,…, *N*) as its columns: ***R****_k_* = [***r****_k_*_,1_,***r****_k_*_,2_, ⋯, ***r****_k_*_,_*_N_*]. Thus, ***R****_k_* can be written as:

(2) $${\text{R}}_{k}={\text{S}}_{k}H_{k}$$

where ***H****_k_* = [***h****_k_*_,1_,***h****_k_*_,2_, ⋯, ***h****_k_*_,_*_N_*] is the *M_k_* × *N* target scattering matrix. The total received signal at all receivers is the superposition of signals from the *K* target cell, which can be written as follows:

(3) $$Y=\sum_{k=1}^{K}{\text{R}}_{k}+\text{W}=\sum_{k=1}^{K}{\text{S}}_{k}{\text{H}}_{k}+\text{W}$$

where ***W*** = [***w***_1_ ***w***_2_, ⋯, ***w****_N_*] is the noise matrix, and ***w****_n_* is the noise vector at the *n*-th receiver.

We consider a statistical MIMO radar with homogeneous and sufficiently separated receivers. Thus, the spatial correlations between the columns of ***H****_k_* can be ignored [7,8]. Likewise, we assume that the noise at different receivers are independent. Specifically, we make the following three assumptions:

Assumption 1 (white noise): The columns of ***W*** are identically and independently distributed (i.i.d.) with distribution *w_n_* ∼ 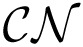
(**0, *R****_w_*) [7,8]. Furthermore, since the temporally white noise can be achieved by prefiltering [4,6], ***R****_w_* can be considered as a diagonal matrix $\sigma_{w}^{2}I$.
  The spatial independence between the columns of ***W*** is satisfied since the thermal noise at different receivers should be independent [7,8]. In the colored noise case, after a whitening filter, a predistortion will be incorporated for transmitted waveform matrix design, and it can be treated as a matrix transformation. In [8], waveform design in colored noise is considered to optimize the performance of detecting a single target.
Assumption 2 (orthogonal waveforms): The signals transmitted by different antennas are orthogonal [6]:
(4) $${\text{s}}_{m}^{H}{\text{s}}_{m'}=\{\begin{array}{cc} \sigma_{m}^{2} & \text{if}\;m=m'\\ 0 & \text{if}\;m\neq m' \end{array}m=m'=1,2,\cdots ,M$$
  where $\sigma_{m}^{2}$ denotes the transmitted energy for transmit antenna *m*.
  Waveforms transmitted by different antennas at different frequency bands can approximately satisfy Assumption 2. In [6], it is also shown that a specific set of frequency spread signals can be orthogonal. In fact, to separate the signals from different targets at the receivers, we do not need to assume orthogonality among all signals. We only need to assume that the waveforms from different 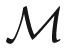
*_k_* are orthogonal. By this, we can apply waveform optimization to each set of 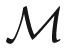
*_k_* for each target *k*. For the moment, we make Assumption 2 for simplicity. Extension to waveform optimization for each set of 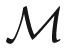
*_k_* will be addressed in future work.
Assumption 3 (target scattering matrix): The columns of ***H****_k_* are i.i.d. with distribution ***h****_k_*_,_*_n_* ∼ 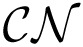
(**0, *R****_H_k__*). Furthermore, considering sufficiently separated transmitters and receivers, the target scattering coefficients are different and independent. Thus, ***R****_H_k__* could be considered as a diagonal matrix ${\text{R}}_{H_{k}}=\text{diag}(\sigma_{H_{k},1}^{2},\sigma_{H_{k},2}^{2},\cdots \sigma_{H_{k},M_{k}}^{2})$ [7].

In a statistical MIMO radar, antennas are sufficiently separated. Thus, *h_m_*_,_*_k_*_,_*_n_*'s can be assumed as independent random variables. Mathematically, the target scattering matrix ***H****_k_* is a full rank random matrix [2]. The diversity of the propagation paths in a statistical MIMO radar can be characterized by this target scattering matrix in Assumption 3. A specific ***H****_k_* can be simulated based on the statistic characteristics of ***H****_k_*. For the moment, we ignore the correlations between the target scattering coefficients for different propagation paths. Extension to a general ***R****_H_k__* case may be addressed in future work.

### 2.2. Multi-Target Detection

Our objective is to employ a MIMO radar to detect multiple targets. To this end, we need to establish a detection model for all targets. Next, we are going to introduce a performance metric on the detection performance of each target.

By Assumption 2, the waveforms transmitted by different antennas are orthogonal to each other. Therefore, each receive antenna can apply *s*_*k*_1__ to the received signal given by Equation (3) to separate the signal from transmit antenna *k*_1_:

(5) $$\begin{array}{ccc} {\text{s}}_{k_{1}}^{H}\text{Y} & = & {\text{s}}_{k_{1}}^{H}\overset{K}{\underset{k=1}{\text{∑}}}{\text{S}}_{k}{\text{H}}_{k}+{\text{S}}_{k_{1}}^{H}\text{W}\\ & = & \underset{M_{k}}{\underset{︸}{\left[s_{k_{1}}^{H}s_{k_{1}},0,\cdots ,0\right]}}H_{k}+{\text{S}}_{k_{1}}^{H}W \end{array}$$

Let ${\text{S}}_{k}^{H}$ collect ${\text{s}}_{k_{1}}^{H}$, ${\text{s}}_{k_{2}}^{H}$, ⋯, ${\text{s}}_{k_{M_{k}}}^{H}$, and thus, we have:

(6) $${\text{S}}_{k}^{H}\text{Y}={\text{S}}_{k}^{H}{\text{S}}_{k}{\text{H}}_{k}+{\text{S}}_{k}^{H}\text{W}$$

Let ${\text{Z}}_{k}={\text{S}}_{k}^{H}\text{Y}$, and the binary hypotheses, 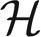
_0_ target-absent and 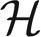
_1_ target-present, are given by:

(7) $$\{\begin{array}{lll} H_{0} & : & {\text{Z}}_{k}={\text{S}}_{k}^{H}\text{W}\\ H_{1} & : & {\text{Z}}_{k}={\text{S}}_{k}^{H}{\text{S}}_{k}{\text{H}}_{k}+{\text{S}}_{k}^{H}\text{W} \end{array}$$

It can be verified that ***Z****_k_* is a sufficient statistics for the optimal Neyman-Pearson detection based on the definition of the sufficient statistics. Here, we omit the proof for brevity.

Next, we investigate the detection performance of all targets. In order to measure the detection performance, we introduce a performance metric of relative entropy, *i.e.*, Kullback-Leibler divergence. It originates from Stein's lemma [29] and approximately characterizes the probability of miss detection. Its advantages have received detailed study in [28]. The relative entropy between *p*_0_(***Z****_k_*) and *p*_1_(***Z****_k_*) is defined as:

(8) $$D_{k}(p_{0}||p_{1})=\int p_{0}({\text{Z}}_{k})\log\frac{p_{0}({\text{Z}}_{k})}{p_{1}({\text{Z}}_{k})}d{\text{Z}}_{k}$$

where *p*_0_(***Z****_k_*) and *p*_1_(***Z****_k_*) are the probability density functions of ***Z****_k_* under hypothesis 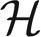
_0_ and 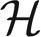
_1_, respectively.

For a given scenario, some transmitters contribute more to the detection performance than others, since they have lower path losses, better angular views of the target and more advantageous wave lengths and polarizations. If each target cell can be illuminated by the proper transmit antennas, which can maximize the received energy, antenna allocation will achieve much more benefit. Therefore, our objective is to select an appropriate transmit antenna subset for each target cell, *i.e.*, to find an optimal allocation scheme to partition the entire transmit antenna set. In order to guarantee the detection performance of each target cell, our objective function is to maximize the minimum relative entropy of all targets. Thus, the target with the minimum relative entropy is detectable, and other targets are also detectable. Here, we assume that the value of low relative entropy is not too much smaller than the value of high relative entropy. If not, we could waste resources on a very faint target, which remains undetectable. Due to the waste regarding the faint target, the optimization could leave other targets as undetectable. Therefore, in realistic scenarios, we can omit the too low relative entropy from the optimization. Here, we also need to be aware that there are other objective functions that we can use. In the case that the detection performance of all target cells needs to be guaranteed, maximizing the minimum relative entropy is one of the effective choices. Other choices may be studied in future work. If the transmitted energy per transmit antenna is fixed, the optimization problem can be formulated as:

(9) $$\begin{array}{ll} \underset{M_{k}}{\max}\underset{k\in K}{\min} & D_{k}(p_{0}||p_{1})\\ \text{s}.\text{t}.\;M_{k} & \subseteq M\\ M_{k} & \cap M_{l}=\phi ,\forall l\in K,l\neq k\\ \sigma_{k,m}^{2} & \leq P_{m},m\in M,k\in K \end{array}$$

where 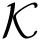 = {1,…, *K*} is the target set and *ϕ* is the empty set. $\sigma_{k,m}^{2}$ is the power of the *m*-th transmit antenna for the *k*-th target, and *P_m_* is the available power of the *m*-th transmit antenna.

## 3. Antenna Allocation Schemes

The analysis in this section is to derive a mechanism for the appropriate allocation of transmit antennas.

### 3.1. Reduced Expression of Relative Entropy

Before we proceed to antenna allocation schemes, we first derive the closed form expression for the relative entropy given in Equation (8) and study its properties. Based on Assumptions 1 and 3, the probability density functions *p*_0_(***Z****_k_*) and *p*_1_(***Z****_k_*) in Equation (8) are written as Equations (10) and (11), respectively.


(10) $$p_{0}({\text{Z}}_{k})=\frac{1}{\pi^{M_{k}N}\det^{N}({\text{S}}_{k}^{H}{\text{R}}_{w}{\text{S}}_{k})}\times \exp\{-\text{tr}[{\left({\text{S}}_{k}^{H}{\text{R}}_{w}{\text{S}}_{k}\right)}^{-1}{\text{Z}}_{k}{\text{Z}}_{k}^{H}]\}$$

(11) $$\begin{array}{ll} p_{1}({\text{Z}}_{k}) & =\frac{1}{\pi^{M_{k}N}\det^{N}({\text{S}}_{k}^{H}{\text{S}}_{k}{\text{R}}_{H_{k}}{\text{S}}_{k}^{H}{\text{S}}_{k}+{\text{S}}_{k}^{H}{\text{R}}_{w}{\text{S}}_{k})}\\ & \times \exp\{-\text{tr}[{\left({\text{S}}_{k}^{H}{\text{S}}_{k}{\text{R}}_{H_{k}}{\text{S}}_{k}^{H}{\text{S}}_{k}+{\text{S}}_{k}^{H}{\text{R}}_{w}{\text{S}}_{k}\right)}^{-1}{\text{Z}}_{k}{\text{Z}}_{k}^{H}]\} \end{array}$$

Substituting Equations (10) and (11) into Equation (8) leads to:

(12) $$\begin{array}{ll} D_{k}(p_{0}||p_{1}) & =N\log\left[\det(I_{M_{K}}+{\text{S}}_{k}^{H}{\text{S}}_{k}{\text{R}}_{H_{k}}{\text{S}}_{k}^{H}{\text{S}}_{k}{\left({\text{S}}_{k}^{H}{\text{R}}_{w}{\text{S}}_{k}\right)}^{-1})\right]\\ & +N\text{tr}\left[{\left(I_{M_{K}}+{\text{S}}_{k}^{H}{\text{S}}_{k}{\text{R}}_{H_{k}}{\text{S}}_{k}^{H}{\text{S}}_{k}{\left({\text{S}}_{k}^{H}{\text{R}}_{w}{\text{S}}_{k}\right)}^{-1}\right)}^{-1}-I_{M_{K}}\right] \end{array}$$

Assuming that the transmit waveform ***S****_k_* consists of *L* identical signal pulses, ***S****_k_* can be expressed as a Kronecker product. We define:

(13) $${\text{S}}_{k}=\left(\begin{array}{c} {\bar{\text{S}}}_{k}\\ {\bar{\text{S}}}_{k}\\ \vdots \\ {\bar{\text{S}}}_{k} \end{array}\right)=1_{L}⊗{\bar{\text{S}}}_{k}$$

where ***S****¯_k_* denotes one pulse of the transmit waveform ***S****_k_*, and **1***_L_* is a *L* × 1 column vector [1,1, ⋯, 1]*^T^*. Based on Assumptions 2 and 3, the autocorrelation matrix of the waveform transmitted by the *k*-th antenna subset and the autocorrelation matrix of the *k*-th target scattering coefficients are both diagonal matrices, which can be written as ${\bar{\text{S}}}_{k}^{H}{\bar{\text{S}}}_{k}=\text{diag}(\sigma_{k,1}^{2},\cdots ,\sigma_{k,M_{k}}^{2})$ and ${\text{R}}_{H,k}=\text{diag}(\sigma_{H_{k},1}^{2},\cdots ,\sigma_{H_{k},M_{k}}^{2})$, respectively. Substituting these two diagonal matrices into Equation (12), after some algebra, the relative entropy can be reduced to a scalar expression:

(14) $$D_{k}(p_{0}||p_{1})=N\sum_{m=1}^{M_{k}}\left(\log(1+\frac{L\sigma_{k,m}^{2}\sigma_{H_{k},m}^{2}}{\sigma_{w}^{2}})-\frac{L\sigma_{k,m}^{2}\sigma_{H_{k},m}^{2}}{\sigma_{w}^{2}+L\sigma_{k,m}^{2}\sigma_{H_{k},m}^{2}}\right)$$

In fact, Equation (14) is simple, since we make those independence assumptions on noise and target scattering coefficients. However, the process of simplification is not the major concern in this paper, and how to simplify a more general model without the assumptions may be addressed in a future study. Next, we can solve the optimization problem (9) via antenna allocation based on Equation (14). Here, we need to be aware that the target scattering coefficients are not known, or we do not need to allocate antennas to improve the detection performance. However, in this problem, the antenna allocation is a dynamic allocation of a set of available antennas. These antennas are allocated to use at each time during a measure period in order to optimize the detection performance. Measure time is partitioned into a sequence of epochs, and one antenna allocation result is employed in one epoch. Our antenna allocation problem refers to the closed-loop solutions to most sensor management problems, *i.e.*, the next allocation is determined while the MIMO radar system is in operation and in view of the results obtained from prior radar measurements in prior epochs [33]. From this point, the antenna allocation optimizes its decision as to how to allocate antennas for the next measurement. We just focus on antenna allocation in one epoch. Particularly, the detection process can be that all transmitters probe each target with some certain number of signal pulses *L̂* first. Thus, based on the received signals, we can obtain a coarse $\hat{L}\sigma_{k,m}^{2}\sigma_{H_{k},m}^{2}$ in the first epoch. Next, we can allocate antennas more flexibly in each epoch to further make a more accurate decision for the detection based on the previous epoch. Thus, we assume that $\sigma_{k,m}^{2}\sigma_{H_{k},m}^{2}$ Equation (14) is obtained in the previous epoch from now on [25,33].

### 3.2. Antenna-Only Allocation Scheme

In this scheme, we only allocate antennas to improve the detection performance. Considering that *D_k_*(*p*_0_‖*p*_1_) is a monotonic nondecreasing function of $\sigma_{k,m}^{2}$, the optimal solution of (9) will be achieved when $\sigma_{k,m}^{2}=P_{m}$, for *m* ∈ 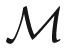 and *k* ∈ 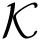. Substituting $\sigma_{k,m}^{2}=P_{m}$ into Equation (14) leads to:

(15) $$D_{k}(p_{0}||p_{1})=N\sum_{m=1}^{M_{k}}\left(\log(1+\frac{LP_{m}\sigma_{H_{k},m}^{2}}{\sigma_{w}^{2}})-\frac{LP_{m}\sigma_{H_{k},m}^{2}}{\sigma_{w}^{2}+LP_{m}\sigma_{H_{k},m}^{2}}\right)=\sum_{m=1}^{M_{k}}Q_{k,m}$$

where $Q_{k,m}=N(\log(1+LP_{m}\sigma_{H_{k,}m}^{2}/\sigma_{w}^{2})-LP_{m}\sigma_{H_{k,}m}^{2}/(\sigma_{w}^{2}+LP_{m}\sigma_{H_{k,}m}^{2}))$ is a constant for given *k* and *m*. If the *m*-th transmit antenna is selected to illuminate the *k*-th target cell, it can contribute at most *Q_k_*_,_*_m_* to the relative entropy of the *k*-th target. If the *m*-th transmit antenna is not selected to illuminate the *k*-th target, it will contribute zero to the *k*-th target, since *Q_k_*_,_*_m_* = 0 for $\sigma_{k,m}^{2}=0$. Thereby, we can introduce a set of binary variables:

(16) $$I_{k,m}=\begin{array}{lll} \{\begin{array}{ll} 1 & \text{If antenna}\;m\;\text{illuminates target}\;k\\ 0 & \mathrm{otherwise} \end{array} & m=1,2,\ldots ,M & k=1,2,\ldots ,K \end{array}$$

to the relative entropy, resulting in the following expression:

(17) $$D_{k}(p_{0}||p_{1})=\sum_{m=1}^{M}I_{k,m}Q_{k,m}$$

Therefore, the problem (9) can be written as:

(18) $$\begin{array}{l} \underset{I_{k,m}}{\max}\underset{k\in K}{\min}D_{k}(p_{0}||p_{1})\\ \text{s}.\text{t}.\;\overset{K}{\underset{k=1}{\text{∑}}}I_{k,m}=1,m\in M \end{array}$$

This type of problem is essentially a minimum makespan scheduling problem in combinatorial optimization algorithms [30]. In [30], the minimum makespan scheduling problem is shown as: given processing times for *n* jobs, *p*_1_,*p*_2_, …, *p_n_* and an integer *m*, find an assignment of the jobs to *m* identical machines, so that the completion time, also referred to as the makespan, is minimized. In the antenna allocation problem, the performance is characterized by the relative entropy. The objective is to maximize the minimum relative entropy. The contributions of antennas are effectively modeled as “processing times” in the makespan scheduling problem, and target cells are effectively modeled as “machines”, which are not identical.

The minimum makespan scheduling problems are classified as 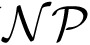-complete in the strong sense [31]. We use a branch-and-bound algorithm and an enhanced factor 2 algorithm to solve this problem, respectively. For a particular scenario, both of these two algorithms only implement antenna allocation over the entire illumination time. Therefore, we put these two algorithms into the same category, called the antenna-only allocation scheme.

#### 3.2.1. Branch-and-Bound Algorithm

This is a classical algorithm for finding optimal solutions of various optimization problems, which was first proposed by Land and Doig in [34]. If computational burden is not considered, a straightforward approach to solve the antenna allocation problem is to enumerate all possible combinations of antennas. Thus, relative entropy function values of all combinations are computed and compared. The optimal solution is the antenna allocation, which leads to the largest function value. However, for problems involving large numbers of antennas, the number of antenna combinations will grow exponentially, which will make the computational burden overwhelming.

The branch and bound algorithm also consists of an enumeration of all candidate antenna allocation solutions. The search process for all antenna combinations is a tree structure, whose nodes are the antenna subsets of all combinations. The step that splits an antenna set into two or more smaller antenna sets is called branching. The step that computes upper and lower bounds for the relative entropy value within a given antenna set is called bounding. A tree node will be discarded, if its upper bound is less than some other node's lower bound. This step is called pruning [34-36]. Using this algorithm, a large number of fruitless candidate solutions can be discarded, which will ease the computational burden to some extent.

To apply the branch-and-bound algorithm in our problem, the problem (18) can be written as the integer programming problem:

(19) $$\begin{array}{ll} \min & d\\ \text{s}.\text{t}. & \overset{K}{\underset{k}{\text{∑}}}I_{k,m}=1,\;m=1,\ldots ,M\\ & I_{k,m}\in \left\{0,1\right\},k=1,\ldots ,K\;m=1,\ldots ,M\\ & -d-\overset{M}{\underset{m=1}{\text{∑}}}I_{k,m}Q_{k,m}\leq 0,\;k=1,\ldots ,K \end{array}$$

Therefore, a branch-and-bound algorithm can be used for this integer programming problem. We need to be aware that branch-and-bound algorithm has exponential worst case complexity, but luckily, it can work with much less effort in some cases. We obtain the optimal antenna allocation result through the branch-and-bound algorithm as a performance bound and compare it with the performance of the proposed enhanced factor 2 algorithm in the experiments.

#### 3.2.2. Enhanced Factor 2 Algorithm

The enhanced factor 2 algorithm is proposed based on the factor 2 algorithm in [30]. The proposed algorithm employs a greedy strategy to arrange the next antenna to illuminate the target cell that has been assigned the least relative entropy so far. The order of arranging antennas is based on the contribution of antennas, *i.e.*, the antenna that can contribute more to the detection performance must have priority. The heuristic scheme is summarized as Algorithm 1.

The relative entropy of each target cell is initialized ℚ*_k_* = 0, *k* ∈ 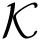. For each *Q_k_*_,_*_m_* corresponding to target *k* and transmit antenna *m*, calculate $r_{k,m}=\frac{Q_{k,m}}{\Sigma_{m=1}^{M}Q_{k,m}}$ as the significance of antenna *m* to target *k*. Out of *k* ∈ 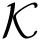 and *m* ∈ 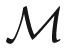, select a pair (*k′*,*m′*) with the maximal *r_k_*_,_*_m_*, and ℚ*_k_*_′_ = ℚ*_k_*_′_ + *Q_k_*_′,_*_m_*_′_. Meanwhile, the antenna is discarded from the remaining antenna set, 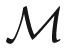 = 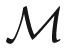
\*m*′. Next, we consider the targets, except *k*′, and thus, we set *r_k_*_′,_*_m_* = − 1(∀*m* ∈ 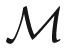). Repeat this step till all targets have been selected once. After that, out of *k* ∈ 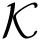, select the one with the minimal ℚ*_k_*. For this cell *k*″, select a transmit antenna *m*″ from the remaining antenna set with the maximal *Q_k_*_″,_*_m_*, and ℚ*_k_*_″_ = ℚ*_k_*_″_ + *Q_k_*_″,_*_m_*_″_. Next, transmit antenna *m*″ is also discarded. Repeat this step till all transmit antennas have been selected, and max min *D_k_* (*p*_0_ ‖ *p*_1_) = min ℚ*_k_*.

Though an exhaustive search algorithm can always achieve a solution if it exists, it is implemented with exponential complexity. The branch-and-bound algorithm has exponential worst case complexity. However, the proposed heuristic enhanced factor 2 algorithm offers significantly reduced complexity, and the performance can be seen in the experiments. Here, we need to note that after max min *D*_k_(*p*_0_ ‖ *p*_1_) is achieved, there can be some antenna *m̃* illuminating the target *k̃* that is not assigned the least relative entropy. Thus, we could reduce the power of these antennas, *i.e.*, $\sigma_{\tilde{k},\tilde{m}}^{2}<P_{\tilde{m}}$ to save resources. This will not influence the antenna allocation result and max min *D_k_* (*p*_0_‖*p*_1_), but it is useful in realistic applications.



**Algorithm 1** Enhanced factor 2 algorithm.
1Initialize ℚ*_k_* = 0, *k* ∈ 

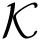
.2Calculate 

${\text{r}}_{k,m}=\frac{Q_{k,m}}{\Sigma_{m=1}^{M}Q_{k,m}}$, *k* ∈ 

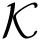
, *m* ∈ 

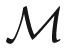
.3**for**
*i* = 1 : *K*  select *k*′ ∈ 

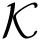
 and *m*′∈ 

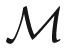
 s.t. max *r_k_*_,_*_m_*;  ℚ*_k_*_′_ = ℚ*_k_*_′_ + *Q_k_*_′,_*_m_*_′_;  *r_k_*_′,_*_m_* = −1 (∀*m* ∈ 

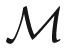
), 

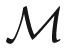
 = 

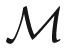
 \ *m*′;**end**.4**while**

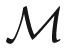
 ≠ *null*  select *k*″, s.t. min ℚ*_k_*;  select *m*″ ∈ 

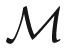
, s.t. max *Q_k_*_″,_*_m_*;  ℚ*_k_*_″_ = ℚ*_k_*_″_ + *Q_k_*_″,_*_m_*_″_, 

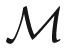
 = 

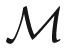

*\ m*″;**end.**5max min *D_k_* (*p*_0_‖*p*_1_) = min ℚ*_k_*.


### 3.3. Antenna-Time Allocation Scheme

Other valid approximation algorithms with high precision for the minimum makespan scheduling problem can be exploited, but we do not focus on these in this paper. We consider that if each target has an opportunity to be illuminated by all transmit antennas, a new appropriate antenna allocation result can be explored. Therefore, we propose an antenna-time allocation scheme to solve the problem from another perspective. Using the antenna-time allocation scheme, antennas are allocated to illuminate different targets with different illumination times.

In order to achieve a proper antenna-time allocation, we propose a method to partition the illumination time based on the illumination probabilities. In particular, the optimal illumination probabilities are obtained first; next, the illumination probabilities can be converted to the illumination time.

Assume that the total illumination time is divided into 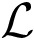 identical units of time. Denote the illumination from antenna *m* to target *k* at the *l*-th unit time by:

(20) $$I_{k,m}(l)=\begin{array}{lll} \{\begin{array}{ll} 1 & \text{If antenna}\;m\;\text{illuminates target}\;k\\ 0 & \mathrm{otherwise} \end{array} & m=1,2,\ldots ,M & k=1,2,\ldots ,K \end{array}$$

where *l* = 1,2,…, 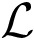. Over each unit time, each transmit antenna selects one of all target cells to illuminate at random with a certain probability. Next, our objective is to obtain the optimal probabilities. We introduce a variable *p_k_*_,_*_m_*(*l*), which denotes the probability of *I_k_*_,_*_m_*(*l*) = 1. For any unit time *l*, it is obvious that $\Sigma_{k=1}^{K}p_{k,m}(l)=1$, and thus, the relative entropy in Equation (17) is treated as an expectation:

(21) $$D_{k}(p_{0}||p_{1})=\text{E}\left\{\sum_{m=1}^{M}I_{k,m}(l)Q_{k,m}\right\}=\sum_{m=1}^{M}p_{k,m}(l)Q_{k,m}$$

The optimal solution of *p_k_*_,_*_m_*(*l*) can be obtained by solving the following linear optimization problem:

(22) $$\begin{array}{ll} \min & d\\ \text{s}.\text{t}. & \overset{K}{\underset{k}{\text{∑}}}p_{k,m}(l)=1,\;m=1,\ldots ,M\\ & 0\leq p_{k,m}(l)\leq 1,k=1,\ldots ,K\;m=1,\ldots ,M\\ & -d-\overset{M}{\underset{m=1}{\text{∑}}}p_{k,m}(l)Q_{k,m}\leq 0,\;k=1,\ldots ,K \end{array}$$

Through linear programming, we can easily obtain the optimal numerical solution of *p_k_*_,_*_m_*(*l*). Observing the problem (22), we can find that *p_k_*_,_*_m_*(*l*) remains constant for *l* = 1, 2,…, 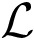, since *Q_k_*_,_*_m_* is a constant for given *k* and *m*. In fact, we assume the target features remain the same over the entire illumination time. Therefore, *p_k_*_,_*_m_*(*l*) can be written as:

(23) $$p_{k,m}(l)=p_{k,m}$$

where *l* = 1, 2,…, 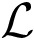. So far, transmit antenna *m* has been arranged to illuminate target *k* at random with a probability *p_k_*_,_*_m_* per unit time. Next, we consider the entire illumination process from antenna *m* to target *k*, which consists of a sequence of illumination units: *I_k_*_,_*_m_*(l), *I_k_*_,_*_m_*(2), …, *I_k_*_,_*_m_*(*L*). This illumination process can be regarded as a stochastic process, which is denoted by *I_k_*_,_*_m_*(*l*). According to Equations (20) and (23), we can obtain:

(24) $$\text{E}\left\{I_{k,m}(l)\right\}=p_{k,m}$$

where *l* = 1, 2,…, 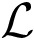. Next, in order to convert the illumination probabilities to the illumination time, we introduce a lemma.

#### Lemma 1

The entire illumination process from antenna *m* to target *k* is a stochastic process with ergodicity:

(25) $$\underset{L\to \infty }{\lim}\frac{1}{L}\sum_{l=1}^{L}I_{k,m}(l)=p_{k,m}$$

Proof. See the Appendix.

Therefore, when the number of unit time is sufficiently large, the time average of the sequence *I_k_*_,_*_m_*(*l*) is the same as the ensemble average. Denote the illumination time from antenna *m* to target *k* by *t_k_*_,_*_m_*. According to Lemma 1, *t_k_*_,_*_m_* can be obtained:

(26) $$t_{k,m}=\sum_{l=1}^{L}I_{k,m}(l)\times t_{\text{unit}}\approx L\times p_{k,m}\times t_{\text{unit}}=t_{\text{total}}\times p_{k,m}$$

where *t_unit_* and *t_total_* are the unit of illumination time and the total illumination time, respectively. Here, we need to be aware that Equation (26) shows a direction to illumination time division, since it partitions illumination time based on the illumination probabilities with some approximations. In reality, the total illumination time cannot be sufficiently long. Moreover, *Q_k_*_,_*_m_* in Equation (21) is related to the unit time. Since *Q_k_*_,_*_m_* does not increase linearly with the unit time, the unit time should not be too short. Though this method is based on approximation analysis, it is not a relaxed solution to the previous 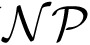-hard allocation problem. In fact, it is an implementable scheme for antenna-time allocation, and the effectiveness of this method can be demonstrated in the experiments.

Next, in order to show an intuitive explanation of the antenna-time allocation scheme, we compare the antenna-time allocation scheme with the antenna-only allocation scheme proposed before. Uniform allocation and time allocation are also used for comparison, both of which employ all antennas to sequentially detect targets one by one. Time allocation optimizes the division of the entire time, while uniform allocation divides the entire time into equal parts for all targets without any optimization.

**Figure 1.**
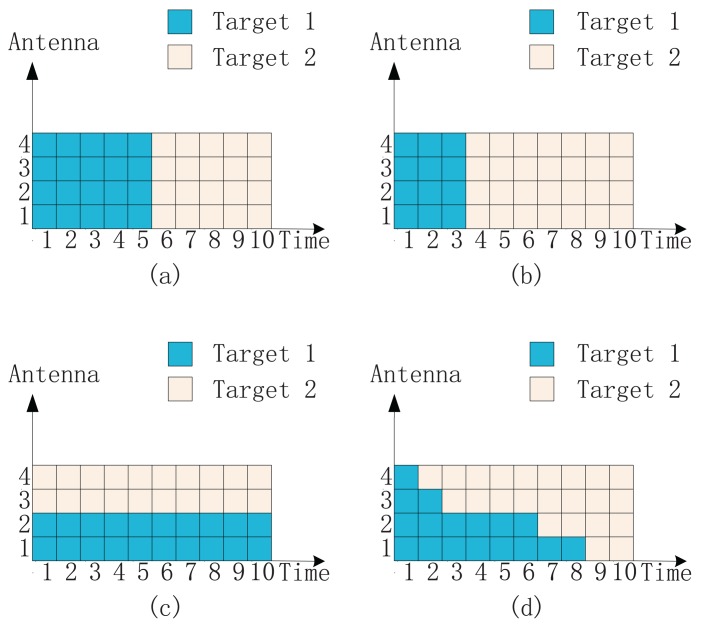
An intuitive explanation of four different illumination schemes: (**a**) uniform allocation; (**b**) time allocation; (**c**) antenna-only allocation and (**d**) antenna-time allocation. The number of antennas is *M* = 4, and the total number of signal pulses is *L* = 10.

Figure 1 shows an intuitive explanation of four different illumination schemes in a two-target detection case. Four transmit antennas are utilized in total, and the illumination time is the length of ten signal pulses. Figure 1a shows the illumination via uniform allocation. The uniform allocation is a general method that employs all four antennas to sequentially illuminate targets one by one. It can just be regarded as the conventional single-target detection scheme. The time for Target 1 equals that for Target 2. Figure 1b shows the illumination via time allocation. It is similar to the uniform allocation. However, the illumination time is divided into two proper parts instead of two equal parts. Figure 1c shows the illumination via antenna-only allocation. We divide all antennas into two proper parts to illuminate two targets simultaneously. Antennas 1 and 2 illuminate Target 1; meanwhile, Antenna 3 and Antenna 4 illuminate Target 2. Figure 1d shows the illumination via antenna-time allocation. Each antenna steers its beam independently towards different targets with different times. The illumination time of Antennas 1–4 for Target 1 occupies 80%, 60%, 20% and 10% of the total time, respectively. Therefore, over most illumination time, we still employ Antennas 1 and 2 to illuminate Target 1; meanwhile, we employ Antennas 3 and 4 to illuminate Target 2. However, it is still possible for both Target 1 and Target 2 to be illuminated by all antennas. In fact, via the antenna-time allocation, we optimize both antenna allocation and illumination time allocation in a more flexible manner.

## 4. Numerical Results and Analysis

In this section, we set scenarios to investigate the detection performance via four different illumination schemes, including a uniform allocation scheme, a time allocation scheme, an antenna-only allocation scheme and an antenna-time allocation scheme. Moreover, the antenna-only allocation scheme contains the branch-and-bound algorithm and the enhanced factor 2 algorithm. Therefore, we use five methods in total to make a comparative analysis. Before simulations, we need to note that in order to allocate transmit antennas for each target cell, we need *M* > *K*. However, the number of targets can be very large in the entire surveillance region. Therefore, we have to detect several times to cover the entire region. For each detection, the number of targets to be illuminated simultaneously should not exceed the number of transmitters, *i.e.*, *M* > *K*.

### 4.1. Experiment Results

**Figure 2.**
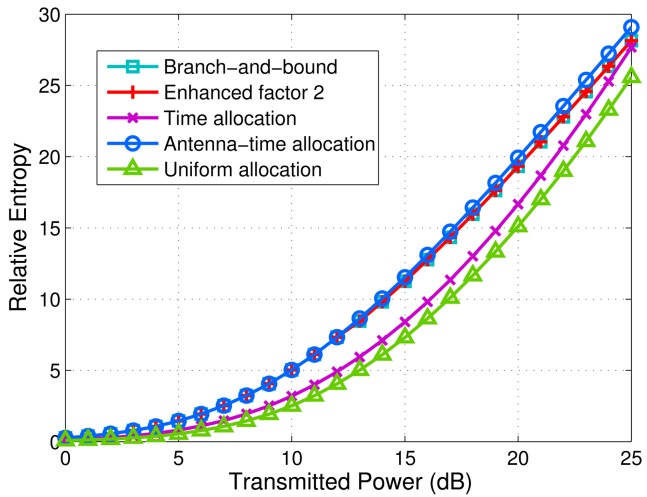
Minimum relative entropy of targets *versus* transmitted power via branch-and-bound algorithm, enhanced factor 2 algorithm, the time allocation scheme, the antenna-time allocation scheme and the uniform allocation scheme. *M* = 4, *N* = 4 and *K* = 2. ***R****_H_*_,1_ = *diag*(5, 0.5, 0.2, 1), ***R****_H_*_,2_ = *diag*(0.01, 2, 1, 0.1) and $\sigma_{w}^{2}=5$.

We set *M* = 4, *N* = 4 and target number *K* = 2. Consider a typical scenario in which ***R**_H_*_,1_ = *diag*(5, 0.5, 0.2, 1), ***R**_H_*_,2_ = *diag*(0.01, 2, 1, 0.1), which means some antennas are more suitable to illuminate a certain target than the others. Particularly, Target 1 is more sensitive to the illumination from Antennas 1 and 4, while Target 2 is more sensitive to the illumination from Antennas 2 and 3. On the whole, the target scattering intensity of Target 1 is higher than that of Target 2. The different sensitivities can be caused by different path losses, angular views, wave lengths and polarizations. Figure 2 shows the minimum relative entropy of all targets *versus* 0–25 dB transmitted power via different schemes. It can be observed that the relative entropy of the schemes that we propose outperforms that of uniform allocation and time allocation. It is obvious that uniform allocation is the worst, since it does not use any optimization. When the transmitted power grows, the relative entropy of the antenna-time allocation scheme gradually outperforms that of the antenna-only allocation scheme, *i.e.*, branch-and-bound algorithm and enhanced factor 2 algorithm. In addition, we can find that branch-and-bound algorithm and enhanced factor 2 algorithm have the same relative entropy value for each transmitted power.

**Figure 3.**
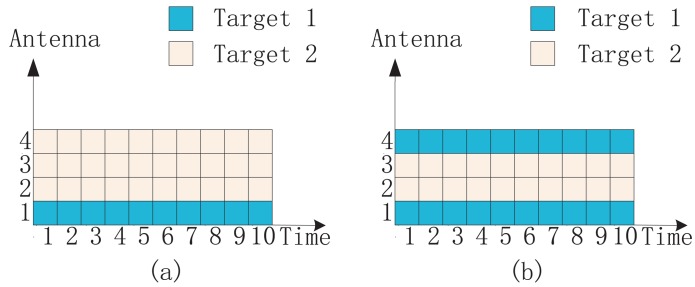
The antenna allocation results via branch-and-bound algorithm in the scenario of Figure 2: (**a**) the antenna allocation with the transmitted power ranging from 0 dB to 12 dB; (**b**) the antenna allocation with the transmitted power ranging from 13 dB to 25 dB. The antenna allocation result via the enhanced factor 2 algorithm is exactly the same as that via the branch-and-bound algorithm.

In fact, the branch-and-bound algorithm and enhanced factor 2 algorithm lead to the same antenna allocation result for each point on the curves in Figure 2. Figure 3 shows the antenna allocation results via the branch-and-bound algorithm. When the transmitted power ranges from 0 dB to 12 dB, the antenna allocation remains constant, which is shown in Figure 3a. It can be seen that only Antenna 1 is employed to illuminate Target 1. When the transmitted power ranges from 13 dB to 25 dB, the antenna allocation is shown in Figure 3b. It can be seen that both of Antenna 1 and Antenna 4 are employed to illuminate Target 1. The antenna allocation result via the enhanced factor 2 algorithm is exactly the same as that via the branch-and-bound algorithm. Therefore, we can just use Figure 3 to show the antenna allocation results via branch-and-bound algorithm and the enhanced factor 2 algorithm.

**Figure 4.**
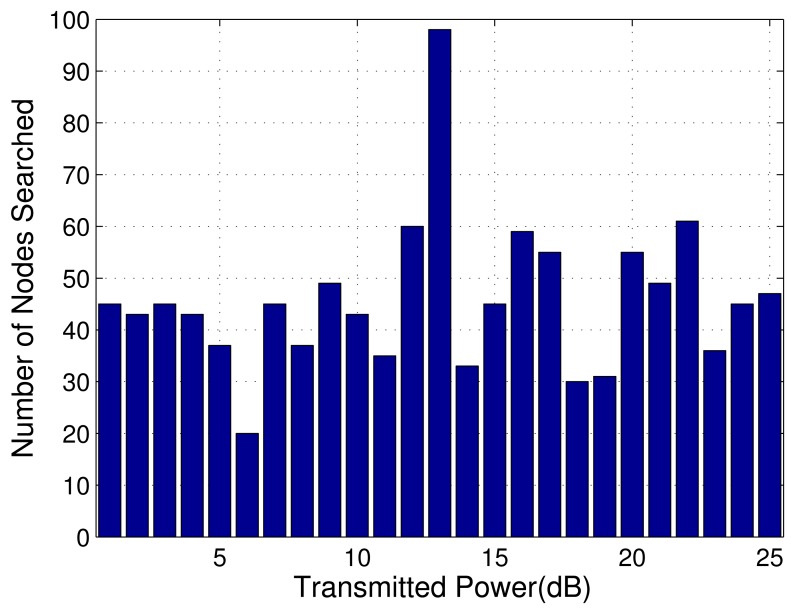
The number of nodes searched via the branch-and-bound algorithm versus transmitted power in the scenario of Figure 2.

Figure 4 shows the number of nodes searched via the branch-and-bound algorithm. It can be observed that the number of nodes is relative large at the transmitted power of 13 dB. Additionally, it has already been shown in Figure 3 that the antenna allocation also changes at this transmitted power. For the computational complexity, this case is worse than the others. Moreover, we can see that in most cases, the branch-and-bound algorithm needs to search relative small number of nodes.

**Figure 5.**
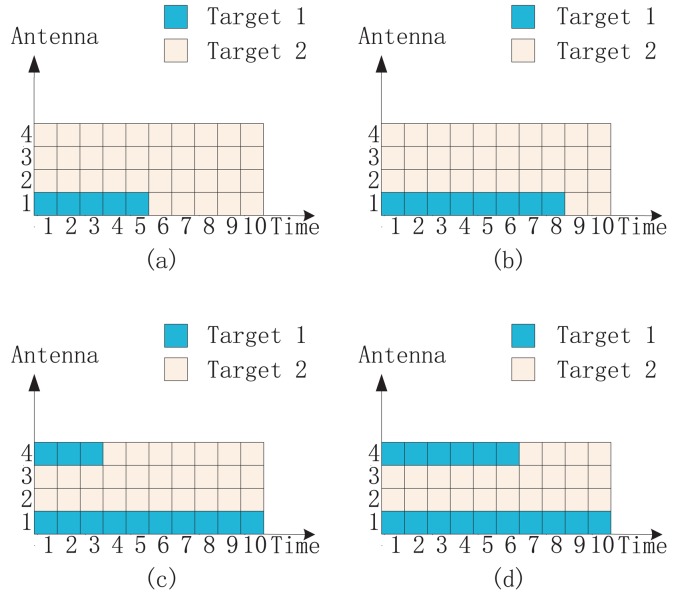
The antenna allocation results via the antenna-time allocation scheme in the scenario of Figure 2: (**a**), (**b**), (**c**) and (**d**) show the antenna allocation results at the transmitted power of 5 dB, 10 dB, 15 dB and 20 dB, respectively.

Figure 5 shows the antenna allocation result in the scenario of Figure 2 via the antenna-time allocation scheme. Figure 5a–d shows the antenna allocation results at the transmitted power of 5 dB, 10 dB, 15 dB and 20 dB, respectively. It can be seen that as the transmitted power grows, the illumination time of Antenna 1 for Target 1 increases unceasingly till it occupies the total time. Antenna 4 does not illuminate Target 1 in the low transmitted power region. As the transmitted power grows, Antenna 4 illuminates Target 1 with ever-increasing time.

**Figure 6.**
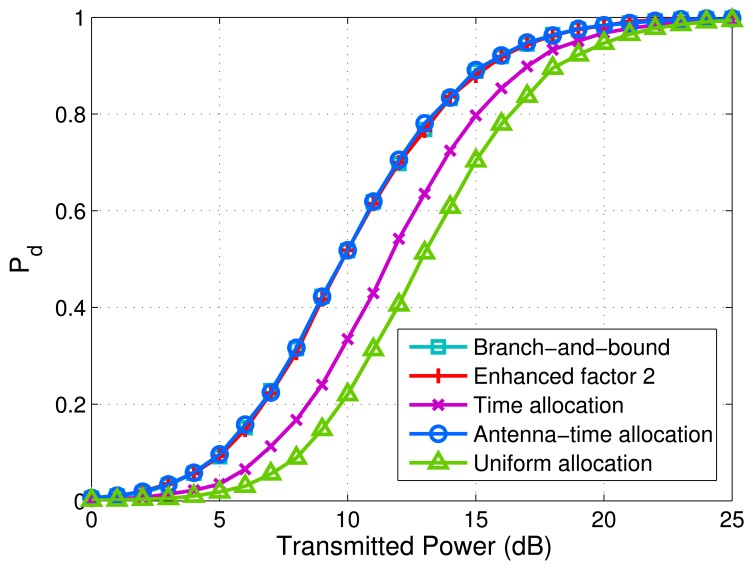
Minimum detection probabilities of targets *versus* transmitted power via branch-and-bound algorithm, enhanced factor 2 algorithm, time allocation scheme, antenna-time allocation scheme and uniform allocation scheme. *M* = 4, *N* = 4 and *K* = 2. ***R****_H_*_,1_ = *diag*(5, 0.5, 0.2, 1), ***R****_H_*_,2_ = *diag*(0.01, 2,1, 0.1) and $\sigma_{w}^{2}=5$. *P_f_* = 0.0001.

Figure 6 shows the minimum detection probabilities of targets *versus* transmitted power via different schemes. The parameters used for simulation in Figure 6 are the same as those in Figure 2. The probability of false alarm is kept constant as *P_f_* = 0.0001. The transmitted power per antenna ranges from 0 dB to 25 dB. In order to observe the detection performance, 10^4^ Monte Carlo trials are executed to achieve the detection probability of each point on the curves. We can find that the branch-and-bound algorithm, the enhanced factor 2 algorithm and the antenna-time allocation scheme almost have the same detection probability for each transmitted power point. All of them can improve detection performance significantly compared with time allocation and uniform allocation.

**Figure 7.**
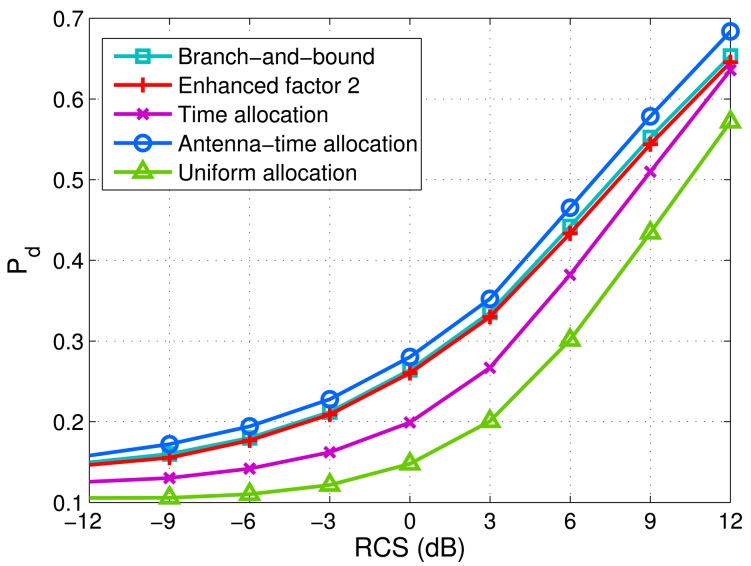
Minimum detection probabilities of targets *versus* target radar cross-section (RCS) fluctuations via five allocation methods. *M* = 4, *N* = 4, *K* = 2, transmitted power *P* = 5 dB and $\sigma_{w}^{2}=5$. *P_f_* = 0.0001.

In order to investigate the influence of different targets on the proposed schemes, we use all illumination schemes to detect different targets in different scenarios. Figure 7 shows the minimum detection probabilities of targets *versus* -12-12 dB radar cross-section (RCS) fluctuations of targets. The statistics of RCS fluctuations can be given by the log-normal distribution model. The variance is from -12 dB to 12 dB. In Figure 7, 10^6^ Monte Carlo trials are executed to achieve the detection probability for each RCS fluctuation variance. It can be observed that two antenna-only allocation algorithms, *i.e.*, the branch-and-bound algorithm and the enhanced factor 2 algorithm, outperform the time allocation scheme.

Over a large range of RCS fluctuations, the antenna-time allocation scheme is the best, while the uniform allocation is the worst.

**Figure 8.**
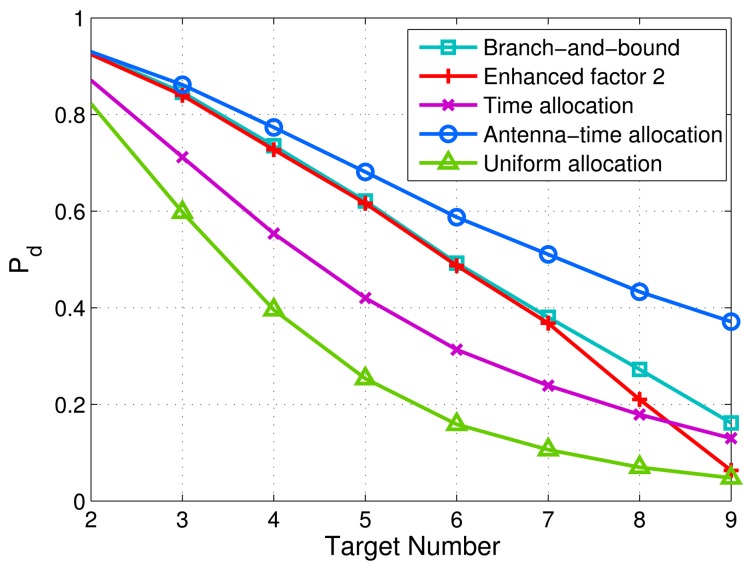
Minimum detection probabilities of targets *versus* target number via five allocation methods. *M* = 9, *N* = 9, $\sigma_{w}^{2}=5$ and 0 dB target RCS fluctuation. *P_f_* = 0.0001. 10^6^ Monte Carlo trials are executed to achieve the detection probability of each point on the curves.

Next, we investigate the influence of the target number on the proposed schemes. We set more antennas: *M* = 9, *N* = 9. The target number varies from two to nine. Figure 8 shows the minimum detection probabilities of targets *versus* target number via different schemes. It can be observed that the proposed antenna-only allocation scheme and antenna-time allocation scheme still have advantages over time allocation and uniform allocation with different target numbers. Moreover, the antenna-time allocation scheme achieves the most robust detection performance when the number of targets is larger.

### 4.2. Analysis

In the experiments above, we have found that the idea of antenna allocation has advantages over the idea without antenna allocation for multi-target detection. In this part, we will show some theoretical analysis.

In the relative entropy Equation (14), we notice that $L\sigma_{k,m}^{2}\sigma_{H_{k},m}^{2}/\sigma_{w}^{2}$ is the ratio of the received signal energy and the noise level at receiver, *i.e.*, SNR. Let $\rho_{k,m}=\sigma_{k,m}^{2}\sigma_{H_{k},m}^{2}/\sigma_{w}^{2}$ denote SNR for one signal pulse; we obtain:

(27) $$D_{k}(p_{0}||p_{1})=N\sum_{m=1}^{M_{k}}\left(\log(1+L_{k}\rho_{k,m})-\frac{L_{k}\rho_{k,m}}{1+L_{k}\rho_{k,m}}\right)$$

where *L_k_* is the pulse number for the *k*-th target. To obtain an intuitive explanation of the relative Equation (27), we assume all *ρ_k_*_,_*_m_*'s for different *k* and *m* are equal, which means all targets are the same isotropic scatterers, and all antennas are the same transmitters with the same path losses, angular views, wave lengths, polarizations, *etc*. Therefore, we drop the subscript of *ρ_k_*_,_*_m_* and obtain:

(28) $$D_{k}(p_{0}||p_{1})=NM_{k}\left(\log(1+L_{k}\rho )-\frac{L_{k}\rho }{1+L_{k}\rho }\right)$$

Observing Equation (28), we notice that *D_k_* (*p*_0_‖*p*_1_) increases linearly with the number of receive antennas *N*. Therefore, when the other parameters are fixed, the number of receivers should be as large as possible to increase *D_k_* (*p*_0_ ‖* p*_1_).

Next, we fix *N* to study the influence of *M_k_* and *L_k_*. Applying the Taylor expansion of log(·) in the small *ρ* region, Equation (28) can be approximately written as:

(29) $$D_{k}(p_{0}||p_{1})\approx \frac{1}{2}NM_{k}L_{k}^{2}\rho^{2}$$

For the large *ρ* region, Equation (28) can be approximately written as:

(30) $$D_{k}(p_{0}||p_{1})\approx NM_{k}\log L_{k}\rho$$

For multi-target detection, *K* denotes the target number. In the scheme without antenna allocation, *i.e.*, time allocation or uniform allocation, we employ all antennas to illuminate targets sequentially Thus, *M_k_* = *M* and *L_k_* = *L*/*K*, where *M* and *L* are the total number of transmit antennas and the total number of signal pulses, respectively Therefore, we have:

(31) $$M_{k}\times L_{k}=\frac{ML}{K}$$

In antenna allocation scheme, we partition all antennas to illuminate *K* targets simultaneously Thus, the average value of *M_k_* is *M*/*K*, and *L_k_* = *L*. We still obtain Equation (31). Therefore, we can see that for each target, the product of the antenna number and the pulse number remains constant, no matter which illumination scheme we use. Thus, Equation (31) can be written as:

(32) $$M_{k}\times L_{k}=C$$

where *C* is a constant for a given *K*. Substituting Equation (32) into Equation (29) leads to:

(33) $$D_{k}(p_{0}||p_{1})\approx \frac{NC\rho^{2}}{2}L_{k}$$

**Figure 9.**
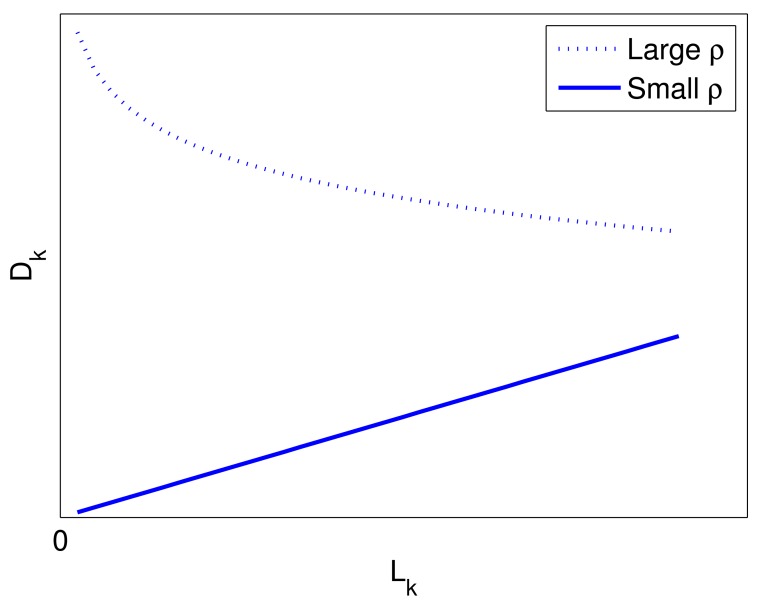
Relative entropy *D_k_ versus* pulse number *L_k_*: the dotted line is the case in the large *ρ* region, and the solid line is the case in the small *ρ* region.

With higher order terms being negligible, we can find that the capacity is in proportion to *L_k_* in the small *ρ* region. Figure 9 shows *D_k_* (*p*_0_‖*p*_1_) in Equation (33) with the solid line. Therefore, in order to obtain a better detection performance in the small *ρ* region, we should select a scheme with larger pulse number *L_k_*, *i.e.*, longer illumination time for a given pulse repetition frequency. Considering *M_k_* × *L_k_* is a constant, we should increase *L_k_* and decrease *M_k_*. Thus, for multi-target detection, we will adopt antenna allocation to illuminate multiple targets simultaneously to lengthen illumination time in low SNR region.

Substituting Equation (32) into Equation (30) leads to:

(34) $$D_{k}(p_{0}||p_{1})\approx \frac{NC}{L_{k}}\log L_{k}\rho$$

Figure 9 shows *D_k_* (*p*_0_‖*p*_1_) in Equation (34) with the dotted line. Considering *M_k_* × *L_k_* is a constant, we can conclude that we should decrease *L_k_* and increase *M_k_* when *ρ* is relatively large. In Figures 2 and 6, the curves of the antenna allocation schemes and the schemes without antenna allocation gradually come close when the transmitted power grows. When the transmitted power is sufficiently strong, the relative entropy of time allocation scheme can be larger than that of antenna allocation schemes. However, using that strong transmitted power, the detection probability has already reached one, no matter which scheme is used. Therefore, it is worthless to increase transmitted power. The experiments also demonstrate that in most cases, antenna allocation schemes outperform the schemes without antenna allocation, as shown in Figures 7 and 8.

## 5. Conclusions

In this paper, a multi-target detection problem for MIMO radar with widely separated antennas is investigated based on relative entropy. In order to explore multi-target diversity, we propose the antenna-only allocation scheme and the antenna-time allocation scheme to implement multi-target detection simultaneously. In the antenna-only allocation scheme, we use a branch-and-bound algorithm and an enhanced factor 2 algorithm, respectively. In contrast to sequential illumination without antenna allocation, simultaneous illumination via antenna allocation can increase illumination time at the expense of decreasing the number of illumination antennas for each target. It is shown that proper antenna allocation outperforms time allocation and uniform allocation over a large range of transmitted power, RCS fluctuations and target numbers. Moreover, the antenna-time allocation scheme can achieve a more robust performance than branch-and-bound algorithm and enhanced factor 2 algorithm for more targets.

## Appendix

In this Appendix, we show the proof of Lemma 1.

The mean and the variance of ${\text{lim}}_{L\to \infty }\frac{1}{L}\sum_{l=1}^{L}I_{k,m}(l)$ can be obtained by Equations (Al) and (A2), respectively:

(A1)
$$\text{E}\left\{\underset{L\to \infty }{\lim}\frac{1}{L}\sum_{l=1}^{L}I_{k,m}(l)\right\}=\underset{L\to \infty }{\lim}\frac{1}{L}\sum_{l=1}^{L}\text{E}\left\{I_{k,m}(l)\right\}=p_{k,m}$$

(A2)
$$\begin{array}{l} \text{E}\left\{{\left|\underset{L\to \infty }{\lim}\frac{1}{L}\overset{L}{\underset{l=1}{\text{∑}}}I_{k,m}(l)-p_{k,m}\right|}^{2}\right\}\\ =\text{E}\left\{{\left|\underset{L\to \infty }{\lim}\frac{1}{L}\overset{L}{\underset{l=1}{\text{∑}}}I_{k,m}(l)\right|}^{2}\right\}-{\left|p_{k,m}\right|}^{2}\\ =\underset{L\to \infty }{\lim}\text{E}\left\{\frac{1}{L^{2}}\overset{L}{\underset{l_{1}=1}{\text{∑}}}I_{k,m}(l_{1})\overset{L}{\underset{l_{2}=1}{\text{∑}}}I_{k,m}(l_{2})\right\}-{\left|p_{k,m}\right|}^{2}\\ =\underset{L\to \infty }{\lim}\frac{1}{L^{2}}\overset{L}{\underset{l_{1}=1}{\text{∑}}}\overset{L}{\underset{l_{2}=1}{\text{∑}}}\text{E}\left\{I_{k,m}(l_{1})I_{k,m}(l_{2})\right\}-{\left|p_{k,m}\right|}^{2}\\ =\underset{L\to \infty }{\lim}\frac{1}{L^{2}}\overset{L}{\underset{l_{1}=1}{\text{∑}}}\overset{L}{\underset{l_{2}=1}{\text{∑}}}\left(R(l_{1},l_{2})-{\left|p_{k,m}\right|}^{2}\right)\\ =\underset{L\to \infty }{\lim}\frac{1}{L^{2}}\overset{L}{\underset{l_{1}=1}{\text{∑}}}\left(p_{k,m}-{\left|p_{k,m}\right|}^{2}\right)\\ =\underset{L\to \infty }{\lim}\frac{1}{L}\left(p_{k,m}-{\left|p_{k,m}\right|}^{2}\right)=0 \end{array}$$

Based on Equations (A1) and (A2), $\lim_{L\to \infty }\frac{1}{L}\sum_{l=1}^{L}I_{k,m}(l)$ equals *p_k_*_,_*_m_* with probability one.
